# Detection of psychosis by mental health care services; a naturalistic cohort study

**DOI:** 10.1186/1745-0179-4-29

**Published:** 2008-12-16

**Authors:** Nynke Boonstra, Lex Wunderink, Sjoerd Sytema, Durk Wiersma

**Affiliations:** 1 GGZ Friesland, Research Department, Leeuwarden, The Netherlands; 2 University Medical Center Groningen, Department of Psychiatry, Groningen, The Netherlands

## Abstract

**Background:**

Detection of psychotic disorders is an important issue, since early treatment might improve prognosis. Timely diagnosis of psychotic disorders depends on recognition of psychotic symptoms and their interpretation. The aim of this study is to examine to what extent reported psychotic symptoms are accounted for in clinical diagnosis.

**Methods:**

The medical files of all patients who had a first contact with one of two mental health care services (N = 6477) were screened for reported psychotic symptoms and subsequent clinical diagnosis. Patients who reported psychotic symptoms and who were diagnosed with a psychotic disorder were followed-up for two years to register prescription of antipsychotic treatment and continuity of care.

**Results:**

In the files of 242 (3.7%) patients specific psychotic symptoms were recorded. 37% of these patients were diagnosed with a non-affective psychotic disorder, 7% with other psychotic disorders and 56% with non-psychotic disorders or no diagnosis at all. About 90% of the patients diagnosed with a psychotic disorder did receive any prescription of antipsychotics, and about 50% were in continuous care during the first 2 years.

**Conclusion:**

Relatively large proportions of patients presenting with psychotic symptoms were diagnosed with a non-psychotic diagnosis or not diagnosed at all. This applies also to patients reporting at least two or more psychotic symptoms. Although we did not verify the appropriateness of clinical diagnosis, these findings are an indication that psychotic disorders may be underdetected. Improving the diagnostic process in mental health care services may be the most obvious way to promote early intervention in psychosis.

## Background

Early detection and intervention of psychotic disorders are important issues in mental health practice and research [1,2]. Different strategies have been developed to improve the detection of first episode psychosis both in the prodromal phase and during the first psychotic episode to minimize the delay of treatment after the onset of psychosis [3,4]. The role of treatment delay has been shown to be an important factor associated with response to antipsychotic treatment, in terms of severity of global psychopathology, positive and negative symptoms, functional outcomes and time to response [5-7]. As a consequence, it was hypothesized that reducing the duration of untreated psychosis would improve outcome [8-13]. Therefore it is essential that patients who report psychotic symptoms at first contact with mental health services are adequately diagnosed and treated accordingly. The APA practice guidelines for the treatment of patients with schizophrenia [14,15] as well as the Dutch guidelines [16] provide recommendations for treatment in every phase of the illness. The most important interventions for first episode psychosis are: accurate diagnostic assessment, initiating antipsychotic medication as soon as it is feasible, and continuation of antipsychotic treatment after response for at least one year in the APA guidelines and at least two years in the Dutch guidelines. The recommended duration of antipsychotic prophylaxis in the Dutch guidelines is one year longer, because relapse rates do not seem to level off during the first years after remission from a first episode [17].

Despite the importance of treatment guidelines, however, implementation has been shown to be difficult to achieve [18-20]. The aim of the study is to examine to what extent reported psychotic symptoms are accounted for in clinical diagnosis and subsequent treatment.

## Methods

### Study design

The design of the study is an administrative inquiry into the diagnostic and daily practice of mental health care services regarding patients who report psychotic symptoms at first contact. The study was conducted in two mental health regions of The Netherlands, with 1.028 million inhabitants on January 1, 2002. The two regions were Friesland (636,000 inhabitants) and Twente (392,000 inhabitants). The total number of inhabitants between 18–45 years of age, representing the at risk population, was 404,909, of which 210,294 (51.9%) were males.

The medical records of all patients between 18–45 years of age who had a first contact with mental health care services in 2002 were screened for reported specific psychotic symptoms and their initial clinical DSM-IV diagnosis. All available documents from the first six months after first contact were taken into account. Patients with at least one of four specific psychotic symptoms were included and followed-up for two years. The psychotic symptoms screened for were the symptoms listed under paragraph A in the schizophrenia section of the DSM-IV: delusions, hallucinations, disorganized speech and grossly disorganized behaviour [21]. After thirty months of follow-up medical files were screened retrospectively for final DSM-IV diagnosis, any prescription of antipsychotic treatment and continuity of care during two year follow up.

## Results

Of 404,909 inhabitants between 18–45 years of age, 6477 (1.6%) were referred to mental health services for a first ever contact in 2002. The medical files of 892 patients were excluded (14%) due to lack of information (526) or availability (366). 5585 medical files were eligible for further research. In the files of 242 patients one or more specific psychotic symptoms were reported; 140 patients (58%) were males. In 182 files delusions were reported, in 90 files hallucinations, in 37 files grossly disorganized behaviour and in 17 files disorganized speech.

As shown in table 1, at baseline 90 patients (37%) were diagnosed according to DSM-IV with a non-affective psychotic disorder (NAPD): schizophrenia, schizophreniform disorder, brief psychotic disorder, schizoaffective disorder, delusional disorder or psychotic disorder not otherwise specified. 17 patients (7%) were diagnosed with other psychotic disorders: organic or substance induced psychotic disorder (n = 6), affective episode with psychotic features (n = 6) or schizophrenia spectrum personality disorder [5]. The other 135 patients were diagnosed with non-psychotic disorders (n = 77, 32%) or were not diagnosed at all (n = 58, 24%). 75 patients (31%) who reported psychotic symptoms had a combination of two or more psychotic symptoms, of whom 45 were diagnosed with a non-affective psychotic disorder (NAPD) and the other 30 patients with other psychotic disorders or a non-psychotic disorder. 16 patients (53%) were diagnosed as a non-affective psychotic disorder sometime during the follow-up period. A combination of at least three symptoms was reported in the records of 9 patients. Of the 167 patients with one reported psychotic symptom, 45 (27%) were diagnosed with a non-affective psychotic disorder.

**Table 1:** Psychotic symptoms per diagnostic group

	NAPD	OPD	Other	None	N
1 sx	45 (27%)	12 (7%)	61 (37%)	49 (29%)	167 (100%)
2 sx	40 (61%)	3 (5%)	15 (23%)	8 (12%)	66 (100%)
3 sx	4 (50%)	2 (25%)	1 (12.5%)	1 (12.5%)	8 (100%)
4 sx	1 (100%)	0	0	0	1 (100%)

NAPD = non-affective psychotic disorder

OPD = other psychotic disorders

Other = other disorders

Sx = symptoms

As shown in table 2, antipsychotic medication has been prescribed anytime during the follow-up period to 158 patients (65%). Of the 90 patients with a non-affective psychotic disorder antipsychotics were prescribed to 81 patients (90%). Of the patients diagnosed with other psychotic disorders (n = 17), 88% received a prescription of antipsychotic medication. Patients diagnosed with other disorders or without any specified diagnosis received a prescription of antipsychotic medication in 46%. Of the 90 NAPD patients, 46 (51%) received continuous care for at least two years.

**Table 2:** Treatment per diagnostic group

	Prescription of antipsychotics	Continuous care	N
NAPD	81 (90%)	46 (51%)	90 (100%)
OPD	15 (88%)	6 (35%)	17 (100%)
Other	33 (43%)	27 (35%)	77 (100%)
None	29 (50%)	14 (24%)	58 (100%)

NAPD = non affective psychotic disorder

OPD = other psychotic disorders

Other = other disorders

## Discussion

The present study, in which more than 5500 medical files were studied from the population of two mental health regions, revealed that many patients presenting psychotic symptoms are not adequately diagnosed. Even when two psychotic symptoms are presented, 25% of patients received a non-psychotic diagnosis or no diagnosis at all. In case one psychotic symptom was stated in the files, the chance of getting a psychotic diagnosis is only 33%. These data strongly indicate that psychosis in mental health care is under detected. We were not able to estimate the prevalence of underdetection of psychosis because of a number of limitations of the study. We were not able to verify clinical diagnosis by a standardized diagnostic procedure, and therefore some of the clinically assigned diagnoses might indeed be appropriate (e.g. in case of PTSS or personality disorder with psychotic symptoms). This study completely relied on reported psychotic symptoms as written in the medical files. Such symptoms might not have been recorded or even noticed and therefore have escaped proper attention. Underdetection of psychotic symptoms may also contribute to underdetection of NAPD. Systematic examination of psychotic experiences at first contact with mental health services may serve as a useful measure to overcome this limitation of our study and of clinical practice. The results did not differ between the two regions with only two mental health care services which were both included in the study, so it is unlikely that local factors played an improtant role or that patients received treatment elsewhere.

90% of the patients clinically diagnosed with a non-affective psychotic disorder received at least one prescription of antipsychotic medication while half of them received continuous care for at least two years. We may conclude that if clinical diagnosis of a non-affective psychotic disorder has been established, proper treatment consequences are more or less assured. This underlines the importance of timely detection of psychotic disorder by mental health care services. Effort should be primarily directed to patients referred to mental health care services in stead of to individuals who do not yet seek help.

## List of abbreviations

APA: American Psychiatric Association; DSM-IV: Diagnostic and Statistical Manual of Mental Disorders; NAPD: non-affective psychotic disorder; PTSS: Post Traumatic Stress Syndrome.

## Competing interests

The authors declare that they have no competing interests.

## Authors' contributions

NB conceived the study, carried out the field study, performed the statistical analysis and drafted the manuscript. LW participated in the design of the study and helped to draft the manuscript. SS supported the statistical analysis and revised the manuscript. DW contributed to the study design and revised the manuscript. All authors read and approved final manuscript.
